# Goldman score, but not Detsky or Lee indices, predicts mortality 6 months after hip fracture

**DOI:** 10.1186/s12891-017-1480-x

**Published:** 2017-04-04

**Authors:** Paula Schmidt Azevedo, David Nicoletti Gumieiro, Bertha Furlan Polegato, Gilberto José Cação Pereira, Igor Almonfrey Silva, Stephan Milhorini Pio, Cacionor Pereira Cunha Junior, Edson Luiz Favero Junior, Sergio Alberto Rupp de Paiva, Marcos Ferreira Minicucci, Leonardo Antonio Mamede Zornoff

**Affiliations:** 1 Internal Medicine Department, Botucatu Medical School, UNESP – Univ Estadual Paulista, Rubião Júnior s/n, ZipCode: 18618-970 Botucatu, SP Brazil; 2 Surgery and Orthopedic Department, Botucatu Medical School, UNESP - Univ Estadual Paulista, Botucatu, Brazil

**Keywords:** Hip fracture, Perioperative assessment, Cardiovascular score, Mortality

## Abstract

**Background:**

Over the past years, several cardiac risk indices were evaluated and modified, including Goldman, Detsky, and Lee scores. The predictive capacity of these scores in hip fracture patients is lacking. Thus, our objective was to compare the Goldman, Detsky, and Lee scores as predictors of mortality in 6 months after hip fracture.

**Methods:**

We prospectively evaluated 80 consecutive patients with hip fractures, over the age of 65 admitted to an orthopedic ward at Botucatu Medical School. Patient demographic information, Goldman, Detsky and Lee scores were recorded. All patients were followed for 6 months after hip fracture, and mortality was recorded. Multiple logistic regression analyses were performed for mortality prediction.

**Results:**

The mortality rate was 23% after a 6-month follow-up period. Patients who died had advanced age and the majority of them were male. They also had lower values of handgrip strength, and higher values of creatinine and urea. In the multiple logistic regression models when adjusted by age, gender, handgrip strength and creatinine, Goldman’s score (OR:3.025; 95%CI:1.022-8.953; p:0.046), but not Detsky (OR:2.328; 95%CI:0.422-12.835; p:0.332) and Lee (OR:1.262; 95%CI:0.649-2.454; p:0.494), was associated with mortality 6 months after hip fracture. Each 1 category increase in Goldman score increased the mortality to more than 3-fold.

**Conclusions:**

In conclusion, our data suggest that Goldman score, but not Detsky or Lee indices, predicts mortality associated with hip fracture at up to 6 months post-injury.

## Background

Approximately 300,000 hip fractures occur annually in the United States. Although the incidence rate varies dramatically across continents, there are expectations that this number will continue to grow exponentially, mainly in developing countries [1–4]. Importantly, the majority of hip fractures occur in the elderly patient population. Emerging evidence suggests that early surgery decreases mortality and minimizes complications secondary to immobilization [5, 6]. However, the perioperative evaluation for hip fracture patients can represent a major challenge, since these patients normally have multiple comorbidities [6, 7]. Thus, extensive preoperative evaluation may lead to worse outcomes.

Usually, the first goal of perioperative assessment is to identify patients at high risk of perioperative cardiac events. It helps define goals for the forthcoming procedure, and also determines whether the procedure is a realistic option [8].

Over the past years, several cardiac risk indices were evaluated and modified, including Goldman, Detsky, and Lee scores [9–11]. Even so, all of the above-mentioned risk indices were developed years ago and since that many changes have occurred in the perioperative management. In addition, data about the predictive capacity of these scores in hip fracture patients is often lacking. Therefore, our objective was to analyze the Goldman, Detsky, and Lee scores as predictors of mortality within the 6 months after hip fracture.

## Methods

This prospective observational study was approved by the Ethics Committee of Botucatu Medical School (3384-2009), and the procedures were in accordance with the Helsinki Declaration of 1975, which was revised in 1983. Written informed consent was obtained from all patients. Eighty patients over the age of 65 admitted to the orthopedic ward with hip fracture who were able to perform the handgrip strength examination correctly were evaluated. The presence of hip fracture related to cancer, and patients who died before our evaluation were excluded. All patients were treated depending on the type of fracture.

For sample size calculation, the following variables were used: 25% of mortality in hip fracture patients, 95% confidence interval and 10% sample error [12, 13]. The minimum sample size required was 72 patients.

After hospital admission, patient demographic information, Goldman, Detsky and Lee scores were recorded. Handgrip strength was performed and blood samples were taken within the first 72 h of admission. All patients were followed for 6 months after hip fracture, and 6 months mortality was recorded.

The Goldman score covers the succeeding items: age higher than 70; myocardial infarction in the past 6 months; S3 gallop or jugular vein distension; important valvular aortic stenosis; rhythm other than sinus, or premature atrial contraction on preoperative electrocardiogram; more than 5 premature ventricular contraction/minute at any time before surgery; intraperitoneal, intrathoracic or aortic surgery; emergency surgery and poor general status. Poor general status was considered when 1 of the following criteria was present: partial pressure of oxygen less than 50 mmHg; potassium lower than 3.0 mEq/L; bicarbonate less than 20 mEq/L; blood urea nitrogen higher than 50 mg/dL; creatinine higher than 3.0 mg/dL; abnormal aspartate transaminases; bedridden from noncardiac causes and liver disease [9].

The Detsky score or American college of physicians score was calculated as previously described. The variables considered were: age higher than 70; myocardial infarction in the past 6 months; myocardial infarction more than 6 months before surgery; unstable and stable angina; pulmonary edema in the last week or ever; critical aortic stenosis; rhythm other than sinus or premature atrial contraction on last preoperative electrocardiogram; more than 5 premature ventricular contraction/minute at any time before surgery; emergency intraperitoneal surgery and poor general medical status [10].

The variables considered for Lee score calculation were: intraperitoneal; vascular or aortic surgery; history of cardiac failure (history, symptoms and signs, X-ray with abnormal cardiac area or congestion, previous echocardiographic study); history of cerebrovascular disease; ischemic heart disease (history, q waves in electrocardiogram, use of nitrates, symptoms or positive test for ischemic disease [11].

Diabetes mellitus definition was based on clinical features and a fasting glucose level of at least 126 mg/dL on two separate occasions or ongoing disease treatment. Systemic hypertension was defined as a systolic blood pressure higher than 140 mmHg and/or a diastolic blood pressure higher than 90 mmHg and smoking was defined as current tobacco use, regardless of the amount of smoking.

### Handgrip strength

Handgrip strengths were measured using a standard adjustable handle (TEC-60; Technical Products; Clifton, NJ, USA). All the measurements were performed for the non-dominant hand, with the elbow supported on the bed, while a competent examiner administered all the tests. Subjects performed three maximum attempts for each measurement, and the best performance of these tests was recorded. During the test, the participant was strongly encouraged to exhibit the maximum strength-power-performance. It was givenone-minute rest period between each attempt to minimize fatigue affects [14, 15].

### Laboratory data analysis/Data analysis and lab reports

Total serum levels of C-reactive protein (CRP), albumin, sodium, potassium, creatinine and urea were measured using the dry chemistry method (Ortho-Clinical Diagnostics VITROS 950®, Johnson&Johnson). The hemogram was performed with a Coulter STKS hematological autoanalyzer.

### Statistical analysis

The data are expressed as the mean ± SD or the median (including the lower and upper quartiles). Statistical comparisons between groups (survivors or not) for continuous variables were executed using Student’s *t*-test for parameters with a normal distribution. If the data were not normally distributed, comparisons between groups were made using the Mann–Whitney test. Fisher’s test or the Chi-square test was used for all categorical data. Parameters that exhibited significant difference in the univariate analysis were included as independent factors in logistic regression models. The only exceptions were variables with high collinearity among them. Therefore, we did multiple logistic regression analyses for mortality prediction, adjusted by age, gender, handgrip strength, and creatinine. Data analysis was performed using SigmaPlot software for Windows v12.0 (Systat Software Inc., San Jose, CA, USA). The significance level was considered to be 5%.

## Results

A total of 80 patients were evaluated, but 5 were excluded because they died before any evaluation. Therefore, 75 patients with a mean age of 79.5 ± 7.8 years were enrolled. Among these patients, 65% were female, and 22.7% died 6 months after fracture repair. The median time from admission to surgery was 6 (4–8) days.

The demographic, clinical and laboratorial data according to 6 months mortality are listed in Table 1. The patient comorbidities were not different between patients who died or survived 6 months after hip fracture. It was identified that patients who died were advanced age and the majority of them, male sex. They also had lower values of handgrip strength, and higher values of creatinine and urea.

**Table 1:** Demographic, clinical and laboratorial data of 75 patients with hip fracture

Variables	6 months mortality		*P* value
	No (*n* = 58)	Yes (*n* = 17)	
Age (yrs.)	78.4 ± 7.2	82.9 ± 8.9	0.04
Female, % (n^o^)	72.4 (42)	41.2 (7)	0.04
Hypertension, % (n^o^)	67.2 (39)	58.8 (10)	0.73
Diabetes, % (n^o^)	22.4 (13)	11.8 (2)	0.50
Smoking, % (n°)	25.6 (20)	35.3 (6)	0.82
Handgrip strength, (kgf)	3.5 (0.0–5.8)	0.0 (0.0–3.8)	0.01
Hemoglobin, (mg/dL)	11.7 ± 2.3	11.3 ± 2.0	0.55
CRP, (mg/dL)	5.3 (3.7–8.2)	8.8 (3.0–21.0)	0.52
Creatinine, (mg/dL)	0.8 (0.7–1.1)	1.1 (0.8–1.6)	0.03
Urea, (mg/dL)	52.0 (35.8–68.0)	81.5 (51.0–121.5)	0.004
Albumin, (g/L)	3.2 (2.9–3.5)	3.1 (2.5–3.4)	0.41
Sodium (mEq/L)	139 (137–140)	139 (135–141)	0.79
Potassium (mEq/L)	4.1 (3.7–4.4)	4.2 (4.0–4.9)	0.09
METs <4, % (n°)	56.9 (33)	82.4 (14)	0.10
TAS, (days)	6.0 (4.0–8.0)	4.0 (3.0–7.5)	0.08

*CRP* C-reactive protein, *METs* metabolic equivalents, *TAS* time from admission to surgery. The data are expressed as the median (including the lower and upper quartiles), as mean ± standard deviation or as proportion

The patients’ classification according to Goldman, Detsky and Lee scores and 6 months mortality is presented in Table 2. There was no difference between scores classification and mortality in hip fracture patients. However, in the multiple logistic regression models when adjusted by age, gender, handgrip strength and creatinine, Goldman’s score was associated with mortality 6 months after hip fracture (Table 3).

**Table 2:** Goldman, Detsky and Lee scores of the 75 patients with hip fracture

Variables	6 months mortality		*P* value
	No (*n* = 58)	Yes (*n* = 17)	
Goldman score, % (n°)			0.11
I	41.4 (24)	23.5 (4)	
II	48.3 (28)	47.1 (8)	
III	10.3 (6)	29.4 (5)	
Destky score, % (n°)			0.16
I	86.2 (50)	70.6 (12)	
II	13.8 (8)	29.4 (5)	
Lee score, % (n°)			0.17
I	32.8 (19)	11.8 (2)	
II	31.0 (18)	35.3 (6)	
III	19.0 (11)	41.2 (7)	
IV	17.2 (10)	11.7 (2)	

The data are expressed as proportion

**Table 3:** Multiple logistic regression models for mortality prediction 6 months after hip fracture

	Odds Ratio	95% Confidence interval	*P* value
Goldman score^a^	3.025	1.022–8.953	0.046
Detsky score^a^	2.328	0.422–12.835	0.332
Lee score ^b^	1.262	0.649–2.454	0.494

^a^Adjusted by gender, handgrip strength and creatinine

^b^Adjusted by age, gender, handgrip strength and creatinine

## Discussion

The aim of our study was to compare the Goldman, Detsky, and Lee scores as predictors of mortality 6 months after hip fracture**.** Our data indicate that Goldman, but not Detsky or Lee, score predicts mortality 6 months after hip fracture.

An important issue is that in our study, hip fracture patients presented 23% of mortality after a 6-month follow-up, similar to other studies [4]. Considering the variables associated with mortality, one may conclude that advanced age, gender (male), renal dysfunction and low handgrip strength were more common among patients with adverse outcome. Therefore, despite advances in the management of hip fracture patients, the short-term mortality after surgery remains elevated.

Another important concern is that it is universally accepted that effective strategies to reduce the risk of complications after surgery must involve cardiac evaluation [16]. Even so, the perioperative evaluation for hip fracture patients can represent a major challenge. In fact, these patients often have multiple comorbidities, and frequent perioperative cardiac over screening [17]. As a result, there was a delay to surgery and the delay was associated with more cardiovascular complications and higher mortality [5, 6, 17].

To avoid unnecessary perioperative cardiac assessment, the first goal of perioperative evaluation is to identify patients at high risk of cardiac events during and after surgery. There are several risk indices used for patient-specific risk quantification. However, none of these have been particularly validated in hip fracture repair [17].

Over the past years, several cardiac risk indices were identified. In 1977, Goldman and col. evaluated risk factors and the Goldman cardiac index was created. This index was updated in 1986 by Detsky et al., which produced a new point system that incorporated patients with recent myocardial infarction, angina, and heart failure [8]. Finally, in 1999, Lee and col. validated the Revised Cardiac Risk Index, a modified version of the original Goldman. This index was designed to predict postoperative myocardial infarction, pulmonary edema, ventricular fibrillation, and complete heart block [16].

Considering the different indices, it is accepted that Lee score is relatively inexpensive and less-time consuming compared with other indices. The Lee index is considered nowadays the most relevant index for predicting cardiac risk in non-cardiac surgery by many clinicians and researchers [16]. On the other hand, it was considered suboptimal for identified patients with multiple risk factors. In addition, recent evidences suggested that Lee index performance was disadvantaged when predicting cardiac events after vascular surgery or predicting death [16]. Therefore, the available data suggested that the performance of cardiac indices might depend on the clinical scenario.

The noteworthy finding in the present study was that Goldman score was the only cardiac index that was an independent predictor of death 6 months after hip fracture surgery. Importantly, each 1 category increase in Goldman score, increased the mortality more than 3-fold. The reasons for this result remain to be elucidated. However, all of our patients were advanced age, associated with different comorbidities. Also, our endpoint was mortality at 6 months. As discussed before, these characteristics were associated with decreased capacity of Lee score to discriminate patients at risk of cardiac events after non-cardiac surgery. Consequently, our data suggest that Goldman score is the best index to predict death 6 months after hip fracture, specifically in the hip fracture scenario.

Finally, we should considerer the main limitations of this study. Firstly, we only included patients from a single medical center. Secondly, our sample size was relatively small. Despite that, we believe that our study adds important information about the performance of cardiac indices as predictors of death after hip fracture.

## Conclusions

In conclusion, our data suggest that Goldman score, but not Detsky or Lee indices, predicts mortality 6 months after hip fracture.

## Abbreviations

CI95%: confidence interval 95%
CRP: C-reactive protein
OR: odds ratio
